# Polymodal Control of TMEM16x Channels and Scramblases

**DOI:** 10.3390/ijms23031580

**Published:** 2022-01-29

**Authors:** Emilio Agostinelli, Paolo Tammaro

**Affiliations:** Department of Pharmacology, University of Oxford, Mansfield Road, Oxford OX1 3QT, UK; emilio.agostinelli@pharm.ox.ac.uk

**Keywords:** Ca^2+^-activated Cl^−^ channels, anoctamin, TMEM16x, scramblases, lipids, gating, Ca^2+^ signalling, SARS-CoV-2

## Abstract

The TMEM16A/anoctamin-1 calcium-activated chloride channel (CaCC) contributes to a range of vital functions, such as the control of vascular tone and epithelial ion transport. The channel is a founding member of a family of 10 proteins (TMEM16x) with varied functions; some members (i.e., TMEM16A and TMEM16B) serve as CaCCs, while others are lipid scramblases, combine channel and scramblase function, or perform additional cellular roles. TMEM16x proteins are typically activated by agonist-induced Ca^2+^ release evoked by G_q_-protein-coupled receptor (G_q_PCR) activation; thus, TMEM16x proteins link Ca^2+^-signalling with cell electrical activity and/or lipid transport. Recent studies demonstrate that a range of other cellular factors—including plasmalemmal lipids, pH, hypoxia, ATP and auxiliary proteins—also control the activity of the TMEM16A channel and its paralogues, suggesting that the TMEM16x proteins are effectively polymodal sensors of cellular homeostasis. Here, we review the molecular pathophysiology, structural biology, and mechanisms of regulation of TMEM16x proteins by multiple cellular factors.

## 1. Introduction to TMEM16x Physiology

The TMEM16x eukaryotic protein family (HUGO gene nomenclature: *Anoctamin*) is composed of 10 paralogues in mammals that share high sequence homology while forming a functionally diverse group of proteins. TMEM16x proteins may (1) form Ca^2+^-activated Cl^−^ channels (CaCCs) (TMEM16A and B) [1]; (2) function as lipid scramblases, which facilitate bidirectional movement of lipids across the cell membranes, possibly in combination with non-selective ion channel activity (TMEM16D, E, F, K and J) [2,3,4,5,6,7,8]; or (3) play additional cellular roles (TMEM16C, G, H) [4,9,10]. Unlike the mammalian TMEM16x family, lower eukaryotes generally have fewer than 10 TMEM16x paralogues [11,12,13].

The TMEM16A and B channels have the highest (~60%) sequence homology within the family [12,14,15,16,17], and present similar electrophysiological properties, including comparable degrees of selectivity and permeability to a range of anions, and sensitivity to intracellular Ca^2+^ [1,18,19,20,21,22,23]. TMEM16A and B channels also share some pharmacological properties; for example, they are modulated in a complex manner by antracene-9-carboxilic acid (A9C) [24,25], and are inhibited by other commonly used Cl^−^ channel blockers—such as 4,4′-diisothiocyano-2,2′-stilbenedisulfonic acid (DIDS) and niflumic acid (NFA)—to a similar extent [22,26,27]. In contrast, a recently identified drug—2-(4-chloro-2-methylphenoxy)-acetic acid 2-[(2-methoxyphenyl) methylene] hydrazide (Ani9)—selectively inhibited TMEM16A, with no significant effect on TMEM16B [28]. The pharmacology of TMEM16x scramblases remains poorly explored, although niclosamide, a therapeutic anthelminthic drug, reportedly inhibits both TMEM16F ion and lipid transport [29], and also inhibits TMEM16A ionic currents [30].

TMEM16x proteins differ in their expression profiles across tissues. TMEM16F and K, for example, are almost ubiquitously expressed in mammalian cells [31]. Other members have more restricted expression profiles and, consequently, play more specific cellular roles. Aspects of the cellular pathophysiology of TMEM16A and some of its paralogues have recently been reviewed [15,32,33,34,35,36,37,38,39,40,41,42]; thus, here we provide a succinct overview of this topic.

TMEM16A is primarily involved in transepithelial Cl^−^ transport [23,43,44,45], and in the regulation of smooth muscle tone [46,47,48,49,50], while TMEM16B participates in the modulation of sensory processes such as olfaction and vision [51,52,53,54,55], as well as the control of the excitability of neuronal and glial cells [45,56,57,58]. The TMEM16D plasmalemmal scramblase [42,59,60] also operates as a non-selective cation channel [6], and can be localised in intracellular membranes during heterologous expression [36]. TMEM16D is primarily expressed in the brain [61] and in endocrine glands, including the adrenal zona glomerulosa, where it may stimulate aldosterone secretion and, thus, contribute to renal control of mean arterial pressure [62]. TMEM16D has been linked to various neuronal disorders [63], such as schizophrenia [64], Alzheimer’s disease [65], and anxiety disorders [66]. TMEM16E and F combine channel and scramblase functions, and have established links to human disease [3,67,68,69,70]. Gain- and loss-of-function mutations in the human *TMEM16E* gene are linked to the bone disease gnathodiaphyseal dysplasia (GDD) [3,68] and muscular dystrophy (MD) [67,68,71], respectively. Mutations in the *TMEM6F* gene are associated with Scott syndrome, a bleeding disorder caused by defective phospholipid scrambling in platelets [59,60,69,70].

Some TMEM16x (TMEM16C, G, and H) proteins play cellular roles other than serving as ion channels or lipid scramblases. TMEM16C interacts with the Na^+^-activated K^+^ (SLACK) channel, increasing the single-channel activity and sodium sensitivity of the SLACK channel [9]. Mutations in the *TMEM16C* gene may lead to autosomal-dominant craniocervical dystonia in human subjects [72]. TMEM16C is also associated with scramblase activity [59], and an early report suggested that it may also have intracellular localisation [36]. TMEM16G is highly expressed in both prostate cancer and normal prostate tissues; it is a proposed candidate for both diagnosis and immunotherapy of prostate cancer [4,73], but the detailed cellular roles of TMEM16G are not fully defined. In some [31,74]—but not all [36,59,60]—published reports, TMEM16G was found to mediate non-selective currents in heterologous systems (FTR and HEK-293 cells), and it was also suggested that it may localise in the endoplasmic reticulum (ER) [36]. TMEM16G also mediated lipid scrambling in a cell line in which the *TMEM16F* gene was deleted [59,60]. TMEM16G may also interact with proteins upregulated during cancer progression [75], including the cellular vesicles staphylococcal nuclease and tudor domain-containing 1 (SND1), the heat shock protein family A (Hsp70) member 1A (HSPA1A), the adaptor-related protein complex 2 subunit beta 1 (AP2B1), and the coatomer protein complex subunit gamma 2 (COPG2); however, the functional significance of these interactions remains undefined [76]. TMEM16H participates in the formation of junctions between the ER and the cell membrane; this process may favour the interaction of proteins and receptors involved in Ca^2+^ release from intracellular stores such as the stromal interaction molecule 1 (STIM1), the inositol 1,4,5-triphosphate (IP_3_) receptor, and the sarco/endoplasmic reticulum calcium ATPase 2 (SERCA2) [10].

While some TMEM16x proteins (such as TMEM16A, B and F) are located in the plasma membrane, TMEM16E, G, H, J, and K are expressed primarily or exclusively in the membranes of intracellular compartments, including the ER; however, there are some conflicting results regarding TMEM16x cellular localisation [2,3,4,10,31,36,74,77,78,79,80,81]. TMEM16K is one of the most studied of the intercellular TMEM16x proteins. TMEM16K is an ER-resident lipid scramblase with non-specific ion channel activity and a dependence on Ca^2+^ and short-chain lipids for optimal activity. The ER membrane, unlike the plasma membrane, has symmetrical lipid distribution. Since many lipids are synthesised on the cytoplasmic side of the ER, scramblases play an important role in the formation of the ER membrane’s symmetrical lipid distribution. TMEM16K has been associated with several cellular phenomena, including spindle formation [82], Ca^2+^ signalling [83], volume regulation [77], and apoptosis [82,83]. The notion that TMEM16K truncations and missense variants lead to autosomal recessive spinocerebellar ataxia type 10 (SCAR10) suggests that incorrect lipid distribution in the ER participates in the pathophysiology of this disease [84,85]. TMEM16J is another TMEM16x with reported intracellular localisation [31,80,81]; however, heterologous expression in HEK293T cells promotes plasma membrane expression, where the TMEM16J works as a cation channel activated by a cAMP-dependent protein kinase A (PKA) [5]. TMEM16J is overexpressed in pancreatic cancer cells [86], in gastrointestinal cancer [87], and in oesophageal squamous-cell carcinoma (ESCC) [81], and is associated with tumour progression [81,86,87].

## 2. TMEM16x Splice Variants

**Figure 1:**
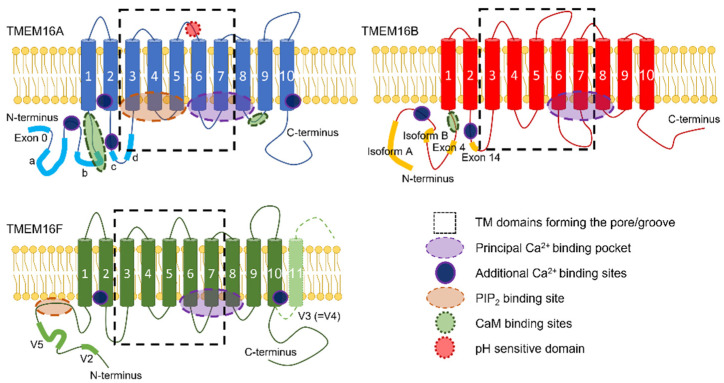
Topological diagram of TMEM16x proteins, showing the position of the alternative spliced exons and a range of functional domains. The boxes (broken lines) highlight the TMs involved in the formation of the ion/lipid pathways in TMEM16x. The approximate position of each splicing variant is labelled using the nomenclature described in the main text. The diagrams also show the approximate position of a range of functional domains, including (1) the principal Ca^2+^-binding pocket, which encompasses conserved acidic residues located in TM6-8; (2) additional regulatory Ca^2+^-binding sites; (3) putative calmodulin (CaM)-binding sites; (4) putative phosphatidylinositol 4,5-bisphosphate (PIP_2_)-binding sites; and (5) the pH-sensitive domain on the extracellular loop between TM5 and TM6 of TMEM16A.

Some *TMEM16x* genes undergo alternative splicing (Figure 1), which leads to the generation of TMEM16x proteins with different functional properties (e.g., sensitivity to Ca^2+^) [7,43,54,55,88,89,90,91,92,93,94,95,96,97,98,99]. The alternatively spliced exons in TMEM16A are termed *a*, *b*, *c*, *d*, and exon 0 (Figure 1). The segment *a* is under the control of an alternative promoter, and codes for an N-terminal cytoplasmic region. The segment *b* distally follows segment *a* in the N-terminus. The segment *c* is located in the first intracellular loop, and codes for a segment of only four amino acids (EAVK). The segment *d* distally follows segment *c* in the first intracellular loop. An alternatively spliced exon, termed exon 0, located upstream of segment *a* in the N-terminus, has also been reported [96]. Finally, another variant has been identified in human interstitial cells of Cajal that lacks exons 1 and 2, as well as part of exon 3, but no specific nomenclature has been attributed to this variant [95,96,97]. The presence of an alternative start codon at position 117 in the primary sequence of abcTMEM16A has been reported. The resulting N-terminal-truncated TMEM16A variant has a restricted tissue expression profile, having only been detected in human testes [98].

Splicing variants have also been reported for TMEM16B, and involve exon 4—within the cytoplasmic N-terminus—and exon 14, which encodes for four amino acids (EAVK) in the first predicted intracellular loop. Alternative starting exons give rise to TMEM16B variants with N-termini of different length (termed A (long) and B (short)). When these two isoforms also lack exon 4 they are termed A_Δ4_ or B_Δ4_, respectively [54,55,91]. Four alternative spliced variants of TMEM16F have been identified [7]. Variant V1 contains 10 transmembrane (TM) segments, and both the N- and C-termini are cytosolic [7]. Variant V2 has an alternative starting exon that gives rise to a shorter N-terminus segment [7]. Variant V3 [7] (also termed V4 in available databases [7]) contains an alternative 3′-terminal exon that results in a TMEM16F isoform with a longer C-terminus portion exposed to the extracellular environment. Variant V5 contains an additional in-frame coding exon in the N-terminus [7,92].

## 3. The Factors Controlling Ion and Lipid Transport in TMEM16x Proteins

TMEM16x proteins are activated in response to an increase in intracellular Ca^2+^ concentration ([Ca^2+^]_i_), typically evoked in response to activation of G_q_-protein-coupled receptors (G_q_PCRs). In addition, the TMEM16A channel and other TMEM16x proteins are controlled by a series of other cellular factors, including pH, anions, hypoxia, ATP, heat, auxiliary proteins and lipid. In virtue of these varied mechanisms of regulation, the TMEM16x proteins effectively constitute polymodal sensors of cellular homeostasis. Here, we review the various cellular mechanisms of regulation of TMEM16x proteins.

### Biophysics of Ion and Lipid Transport

The amplitude of a whole-cell ion channel current (*I*) is determined by the product of the number of channels in the membrane (*N*), the channel-open probability (*P_o_*), and the single-channel current (*i*) as *I* = *NP_o_i*. The magnitude of the single-channel current (i.e., the current flowing through the open pore) is determined by the ionic electrochemical gradient and the chemical characteristics of the permeation pathway (pore conductance). The fraction of time the channel spends in the open state defines the *P_o_*. 

For the TMEM16x channels, the *P_o_* is primarily regulated by changes in [Ca^2+^]_i_ and voltage (V_m_), since binding of Ca^2+^ to a specific high-affinity site, which is favoured at depolarised V_m_, leads to channel opening (see Section 4.1). Other factors, such as pH, ATP, heat etc., also modulate TMEM16x channel gating. *N*, the number of channels in the membrane, is the result of gene expression and protein trafficking, processes that are dynamically regulated and can be altered in disease. The regulation of *N* typically occurs in minutes to days, while the channel response to changes in Ca^2+^ and V_m_ is much more rapid. Furthermore, TMEM16x splice variants vary in their functional properties; thus, regulation of TMEM16x splicing offers an additional means of modulation of whole-cell current properties (kinetics, Ca^2+^ sensitivity). The equation above implies that permeation (*i*) and gating (*P_o_*) are separate factors. However, this is an approximation, since for the TMEM16x channels the permeating anion may also influence channel gating and apparent Ca^2+^ sensitivity. For example, activation of TMEM16A and B is facilitated by anions with high permeability, or by an increase in extracellular Cl^−^ [19,100], suggesting that the conductive pore and Ca^2+^-dependent gating are coupled. It has been proposed that bulkier anions favour the channel-open state by increasing the mean open time via a mechanism similar to the ‘foot in the door’ produced by quaternary ammonium ions on K^+^ channels [101,102,103].

A conceptually similar equation to the one described above for the TMEM16x current could be used to describe TMEM16x scramblase function. For scramblases, the equivalent of *i* is given by the lipid transport occurring through a single TMEM16x scramblase protein per unit of time, *P_o_* is the fraction of time the scramblase spends in the ‘lipid-conductive’ mode, and *N* is the number of TMEM16x scramblases expressed on the membrane. The cellular factors (reviewed below) that control TMEM16x primarily affect the *P_o_*; however, it has been reported that ATP may modulate *i* of TMEM16A via phosphorylation of a serine in the third intracellular loop [104]. Some structural domains in TMEM16A and B that control their expression on the plasma membrane (which influences *N*) have also been identified [18].

## 4. Overview of the Structure–Function Relationship in TMEM16x Proteins

The structures of the mammalian TMEM16A channel [105,106,107,108] and fungal [109,110,111,112] or mammalian TMEM16x lipid scramblases [2,113,114] have been experimentally resolved via X-ray crystallography and/or cryo-electron (cryo-EM) microscopy. Here, we offer a concise summary of the relationship between structure and function in TMEM16x proteins. Residues are defined by the International Union of Pure and Applied Chemistry (IUPAC) one-letter codes, and their position in the primary sequence is given alongside the splice variant utilised in each cited study (when this was specified in the original publications).

**Figure 2:**
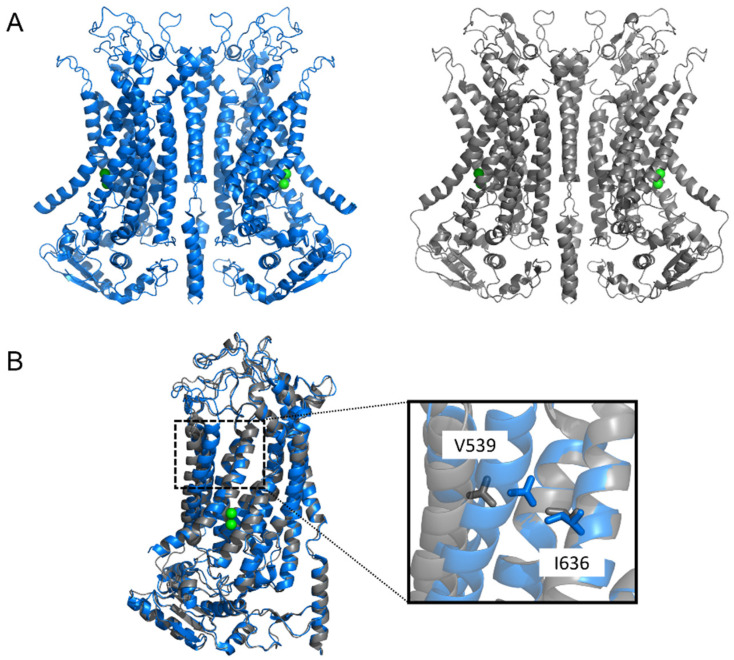
Structural alignment of the experimentally determined Ca^2+^-bound structure of TMEM16A channel, and a model of the TMEM16A channel in the open state. (**A**) **Left**: Lateral view of the dimeric Ca^2+^-bound TMEM16A cryo-EM structure (PDB ID: 5OYB, blue). **Right**: TMEM16A open-state model (from [122], grey). The Ca^2+^ ions are shown in green. (**B**) **Left**: Single monomer of the Ca^2+^-bound TMEM16A cryo-EM structure (5OYB, blue), aligned with the open-state model (grey) [122]. The Ca^2+^ ions are shown in green. **Right**: Magnification of the gate-forming pore residues (V539 and I636, from [122]), in the Ca^2+^-bound TMEM16A cryo-EM structure (5OYB, blue), and of the TMEM16A open-state model (grey) [122], as indicated.

The X-ray structure of the *Nectria haematococca* TMEM16 (nhTMEM16)—a scramblase with non-selective channel activity—demonstrated a dimer arranged in a bi-lobal ‘butterfly’ fashion, with each subunit presenting 10 TM α-helices [109] (Figure 1 and Figure 2). Each monomer has a hydrophilic, membrane-spanning groove that provides a pathway for lipid headgroups to move across membranes. This lipid-scrambling mechanism has been reproduced in silico via molecular dynamics (MD) [115,116]. The structure of another fungal TMEM16 protein—the *Aspergillus fumigatus* afTMEM16 [110]—in conjunction with the structures of nhTMEM16 [111], murine TMEM16F [113,114], and human TMEM16K [2], demonstrated a range of conformations that these scramblases can assume. These involve movements of helices near the lipid-scrambling pathway enabling a range of activation states, potentially triggered by diverse stimuli such as membrane thickness, lipid composition, post-translational modification, or binding of cofactors.

Lipid scramblase activity critically depends on both the membrane lipid composition and membrane thickness. For example, lipid scrambling by TMEM16K is greatly augmented in the presence of shorter chain lipids, which form thinner bilayers, similarly to the ER membrane [2,79]. Continuous scrambling occurs in the ER membranes to help to redistribute lipids that are synthesised on the cytoplasmic side of the ER membrane. In contrast, the plasma membrane has a highly asymmetrical lipid distribution, which is dissipated under specific conditions, such as apoptosis. Thus, plasmalemmal scramblases, such as TMEM16F, need to be tightly regulated [7,8,60,70]. The dependence of TMEM16K on membrane thickness might provide a safety mechanism to preclude this protein from dissipating the plasma membrane asymmetry in case of aberrant trafficking leading to TMEM16K insertion to non-ER membranes [2].

**Figure 3:**
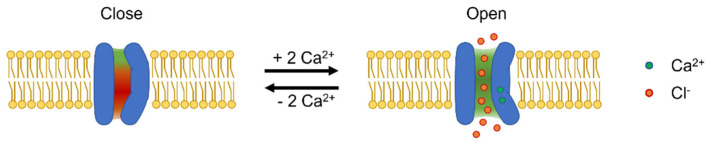
TMEM16A close and open conformation diagram. Diagrammatic representation of a single TMEM16A channel pore and related gating mechanisms. Ca^2+^ binding induces opening of the steric gate in TM6 and attenuation of the electrostatic gate. The movement of the TM6 helix during gating is represented as a tilt of the inner portion of the pore and opening of the outer pore. The green and red backgrounds depict positive and negative electrostatic potentials in the pore, respectively.

Like the TMEM16x scramblases, the TMEM16A channel is a homodimer, and each monomer forms an independent pore [117,118] (Figure 2 and Figure 3). Experimentally determined structures of murine TMEM16A revealed two transmembrane α-helices (TM4 and TM6), effectively blocking the top of what constitutes the scramblase groove in scramblase homologues [105,107,108] (Figure 1). This results in the formation of a protein-enclosed ion-conductive pore in TMEM16A that is for the most part shielded from the membrane, but which might be partly accessible to lipids on its intracellular side [105,107,108], where the detachment of TM4 and TM6 forms a funnel-shaped vestibule that has portions directly exposed to the cytoplasm and the lipid bilayer. The shape of this ion-conductive pore resembles the shape of an hourglass. The hydrophilic membrane-exposed cavity in TMEM16x scramblases has evolved in TMEM16x channels to form an aqueous pore mostly shielded from the membrane lipids (Figure 1). The intracellular region connecting TM4 and TM5 plays a role in lipid scrambling in TMEM16F (termed the ‘scramblase domain’) [8]. Transfer of the TMEM16F or TMEM16E scrambling domains to TMEM16A confers scrambling activity to TMEM16A [8,119,120].

### 4.1. Gating Mechanisms

Each TMEM16A monomer has a principal high-affinity Ca^2+^-binding pocket for two Ca^2+^ ions; binding of Ca^2+^ at this site triggers channel opening [105,107] (Figure 1, Figure 2 and Figure 3). Each pore possesses a steric gate constituted by an intracellular portion of the sixth transmembrane α-helix (TM6) [107,121]. A hinge-point formed by glycine at position 640 (in aTMEM16A) enables a conformational rearrangement of this gate in response to Ca^2+^ binding [107,121] (Figure 3). Alanine substitution of I637 or Q645 (in aTMEM16A) stabilises the TM6 gate in the open state, which results in channel opening in the absence of intracellular Ca^2+^ at positive V_m_ [121,122,123]. The principal Ca^2+^-binding pocket encompasses a series of negatively charged residues [123], namely, E650, E698, E701, E730, and D734 (in aTMEM16A); except for E698, these residues are highly conserved in the various TMEM16x paralogues and homologues [124,125,126,127,128,129]. The proximity of these residues to the permeation pathway means that the vacant Ca^2+^-binding pocket provides an electrostatic barrier to anion permeation (termed the ‘electrostatic gate’). Binding of Ca^2+^ at this site screens the negative charge density of the Ca^2+^-binding pocket, thus producing attenuation of the electrostatic gate [123].

The existence of a low-affinity Ca^2+^-binding site in an intracellular domain of the channel has also been suggested [117,118], but the residues forming this site and its functional role are not fully defined. The recently solved TMEM16K [2] and TMEM16F [113,114] structures indicate a potential additional binding site for Ca^2+^ at the interface between the two TMEM16x monomers, and involving residues in TM2 and TM10 (Figure 1). This site is also found in TMEM16A, and mutations of key residues (i.e., E425, K428, D879, and D884 in aTMEM16A) reduce the channel activation in response to [Ca^2+^]_i_ elevation [130]. Furthermore, an EF-hand-like region is also present at the N-terminus of TMEM16A and B [131] (Figure 1). Electrophysiology experiments demonstrated that TMEM16F proteins engineered to harbour this EF-hand-like domain have enhanced Ca^2+^ sensitivity [132]. In addition, the intracellular loop between TM2 and TM3 encompasses a segment composed of four glutamates in succession, adjacent to the spliced segment *c* of TMEM16A (exon 14 in TMEM16B) (Figure 1). This region is known to modulate Ca^2+^ sensitivity, and may bind Ca^2+^ directly in both TMEM16A and B channels [100,133,134].

For the TMEM16x scramblases such as human TMEM16K, afTMEM16, and nhTMEM16, Ca^2+^ binding triggers widening of the outer region of the groove to enable lipid scrambling [2,110,135]. An analogous gating movement is observed in the outer pore of the TMEM16A channel (Figure 3) [106,122,136]. This gating structure is composed of hydrophobic residues in TM4 and TM6, such as I550, I551, and I641 in acTMEM16A [106,136,137], and V539 and I636 in aTMEM16A [122,137] (Figure 2). Thus, TMEM16x channels and scramblases may share a common Ca^2+^-dependent mechanism that widens the outer pore/scrambling groove. The observation that single-point pore mutations confer scramblase activity to TMEM16A [138] is consistent with the idea that the lipid- and ion-permeation pathways in TMEM16x proteins may share a similar overall structural arrangement. These single-point mutations are (1) substitution of V543 in acTMEM16A into a threonine or serine (i.e., the nhTMEM16 and TMEM16F residues found in the position corresponding to V543 of TMEM16A), or (2) substitution of K588 in acTMEM16A into asparagine (i.e., the equivalent residue found in nhTMEM16) [138].

Intracellular Ca^2+^ is mandatory for TMEM16F activity [60,113,128], while afTMEM16 [139], nhTMEM16 [109] and, to a lesser degree, human TMEM16K [2], show some constitutive scrambling activity even in the absence of intracellular Ca^2+^. This basal activity might be ascribed to a high degree of mobility for TM3 and TM4 in fungal scramblases, and multiple flexible residues (such as P332 and G339 for nhTMEM16) may be involved [110,111]. The possibility that scramblase activity in the absence of Ca^2+^ is due to the presence of other cofactors cannot be ruled out [3,8,138].

### 4.2. Ion and Lipid Conduction Pathways

The ion conduction pathway in TMEM16A consists of a funnel-shaped intracellular vestibule that narrows to a tight pore at the extracellular part of the membrane [105,108]. Permeating ions need to shed their hydration shell as they pass through the narrow section of the pore; thus, the anion selectivity of TMEM16A follows a lyotropic sequence [1,17,140]. The relative anion permeability of TMEM16A measured under bi-ionic conditions is SCN^−^ > NO_3_^−^ > I^−^ > Br^−^ > Cl^−^ > F^−^ > gluconate [1,17,18,141]. Similar anion selectivity and permeability sequences have also been reported for TMEM16B and F [18,19,22,54,142,143,144,145].

The TMEM16A pore is amphiphilic and contains charged, polar, and apolar residues. Anion permeation in TMEM16A requires Cl^−^ binding to a series of positively charged residues within the pore, including K584, R617, and K641 (in aTMEM16A) [108,117,118,122,146]. Mutations of pore residues R573 and K540 in TMEM16B (corresponding to R617 and K584 in aTMEM16A, respectively) lead to alterations in the ion selectivity and Ca^2+^ sensitivity of the TMEM16B channel, emphasising that TMEM16A and B channels share similar pore structures and permeation mechanisms [18,21].

TMEM16F channels are poorly selective for anions and cations. Whole-cell recordings of TMEM16F currents in a heterologous system (HEK-293 cells) demonstrated a slightly higher permeability to Cl^−^ than to Na^+^ [142,144,147,148,149,150,151,152]; however, recordings obtained in excised inside-out patches (HEK-293 cells) revealed that TMEM16F was more permeable to cations than to anions [113,128,143]. Thus, the electrophysiological properties of TMEM16F are altered during inside-out recording, possibly because patch excision may cause disruption of the interaction of TMEM16F with the cytoskeleton and/or cytoplasmic factors [153]. The residues lining the pores of TMEM16F are not fully defined. It is noteworthy that K584 in aTMEM16A corresponds to Q559 in TMEM16F. The anionic selectivity of TMEM16A, relative to Na^+^, is reduced by glutamine substitution of K584, while the cationic selectivity and scramblase activity of TMEM16F are reduced by lysin substitution of Q559 [113,114,128,143,145,154].

As noted above, in TMEM16x scramblases, the TM4 and TM5 form a groove that enables lipid translocation. The region that constitutes the scramblase domain (see Section 4) can accommodate the headgroups of different sized lipids, but the narrow neck (the narrowest point of the hourglass shape) needs to widen in order to enable lipid scrambling [59,60,109,139]. MD simulations of nhTMEM16 demonstrated that lipids interact with charged and polar amino acids in the intracellular (e.g., E352, K353, R505, N378, Q374) and extracellular vestibules (e.g., E318, E313, R432, T333, Y439) [115,135,138]. It has been proposed that these interactions trigger conformational rearrangements that open the narrow neck [135], enabling lipid scrambling through a so-called ‘credit card mechanism’ [155]. Polar uncharged residues (e.g., T381, S382, T340) within the groove pathway are also important for lipid translocation, and mutations of these residues result in impaired lipid trafficking [120,138]. The E529 and K530 residues in V2-TMEM16F (conserved in TMEM16C, D, and E, and corresponding to E352 and K353 of nhTMEM16, respectively) are located deeper in the membrane compared to nhTMEM16, and only K530, but not E529, is essential for PS exposure [120]. Moreover, Y439, which is important for scrambling in fungal homologues, is not conserved in mammalian TMEM16x scramblases. Thus, despite a low sequence similarity between distant family members, such as TMEM16F and nhTMEM16, there is a remarkable conservation of function, indicating that scramblase activity tolerates sequence divergence [156,157].

A series of mechanisms can explain the combined ion channel and scramblase function of some TMEM16x proteins; these include (1) the ‘alternating pore–cavity’ model, which implies that ions permeate through an intermediate, semi-closed conformation of the lipid/ion permeation pathway that is nonconductive to lipids, but permeable to ions [113], and (2) the proteolipidic pore model, which implies that ions and lipids are transported through the fully open cavity lined with lipid headgroups [158]. These propositions have been tested in silico using MD simulations of fungal nhTMEM16, human TMEM16K, and murine TMEM16F. The results of these investigations suggest that the intermediate state of the permeation pathway does not allow the passage of ions and lipids [159]. Thus, Ca^2+^ binding may promote full opening of the pore/groove, enabling both ion conduction and lipid scrambling [138,159].

## 5. Mechanisms of Modulation of TMEM16x Proteins’ Function

### 5.1. Ca^2+^ and Other Divalent Cations

**Figure 4:**
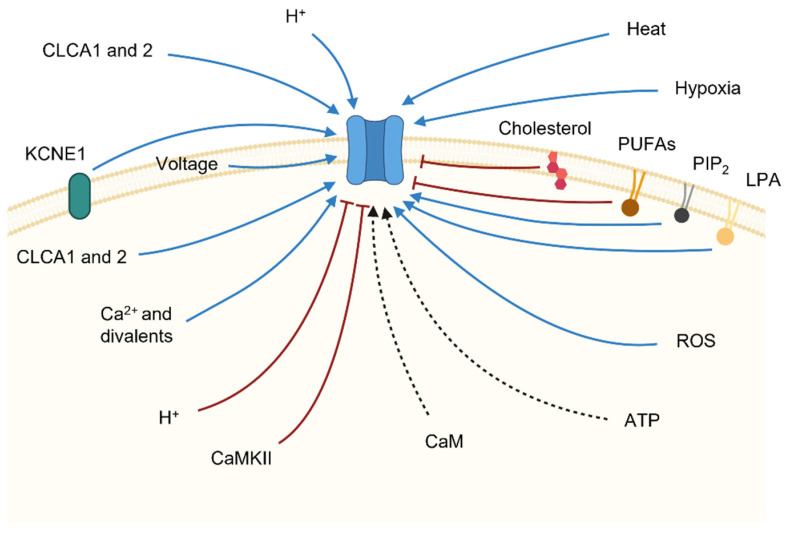
Summary of the principal factors that control TMEM16x function. Schematic representation of various physiological modulators of TMEM16x activity. A TMEM16x protein in the plasma membrane of a generic cell is shown in blue. Factors that control TMEM16x activity are connected to the protein with red or blue lines to denote inhibitory or stimulatory influences, respectively. The black dashed arrows indicate the modulations for which an underlying mechanism has not yet been fully defined and/or conflicting evidence has been proposed.

An increase in [Ca^2+^]_i_ is the prime stimulus for the activation of TMEM16x proteins (Figure 4, Table 1). The response of native CaCCs to Ca^2+^ is characterised by a half-maximal effective concentration (EC_50_) in the range of 0.2–5 µM [14,160]. The Ca^2+^ response curve for native CaCC currents has a Hill coefficient > 1, which is consistent with the estimated binding of 2–3 Ca^2+^ required for channel activation [161,162,163], and the recent structural findings showing two bound Ca^2+^ ions in each TMEM16x monomer [105,107] (Figure 2). The Ca^2+^ sensitivity of cloned TMEM16A and B channels is in the same range as that of native CaCCs, with EC_50_ values depending on the clone species, splice variant, and recording conditions (e.g., the configuration of patch-clamp technique, or the V_m_ at which the recording was made) [17,18,22,54,100]. Intracellular Ca^2+^ activates TMEM16x channels in a V_m_-dependent manner; the Ca^2+^ sensitivity increases proportionally to the degree of V_m_ depolarisation [1,18,127,133]. This is likely a direct consequence of the location of the principal Ca^2+^-binding pocket within the membrane-spanning region of TMEM16x proteins—an arrangement that favours intracellular Ca^2+^ binding at depolarised V_m_ [105]. As a result, the current versus V_m_ relationship for either TMEM16A or B channels is outwardly rectifying at low Ca^2+^ (<~1 μM), but becomes almost linear at high Ca^2+^, when Ca^2+^ binding occurs at depolarised and hyperpolarised V_m_ [18,22,164]. The possibility of V_m_-dependent conformational changes taking place after Ca^2+^ binding has also been suggested [106,157].

TMEM16A currents can be activated in the absence of intracellular Ca^2+^ at highly depolarised V_m_ (>~120 mV) [25,100] (Figure 4, Table 1). Unlike other V_m_-dependent channels—such as V_m_-gated Na^+^ (Nav), Ca^2+^ (Cav), and K^+^ (Kv) channels—the TMEM16x proteins lack any specific V_m_-sensing domains, comprising a series of positively charged residues. Consistently, no gating currents (which reflect the movement of the voltage sensors of V_m_-gated channels) have been detected in TMEM16A channels during recordings in 0 [Ca^2+^]_i_ and in the presence of blockers to prevent the ionic currents [165]. The intrinsic V_m_ dependence in TMEM16A channels may be the result of allosteric activation of the channel by extracellular Cl^−^ [165]. TMEM16x channels also lack fast inactivation, which is typical of some V_m_-gated channels such as Nav, Cav, and some Kv. However, prolonged exposure to high [Ca^2+^]_i_ induces TMEM16x channel desensitisation [21,22,54,105,129,141,145,164] via a mechanism that might involve PIP_2_ depletion [164,166,167,168].

TMEM16F has an EC_50_ for Ca^2+^ of ~1 µM for scrambling activity [113]. The reported Ca^2+^ sensitivity of the TMEM16F channel function varies dramatically depending on the patch-clamp configuration used, with the reported EC_50_ equalling ~5–30 µM or 10–100 µM during inside-out [113,128,143,145] or whole-cell [132,142,144,145] recordings, respectively. These differences might be due to loss of cellular factors (e.g., PIP_2_) during patch excision [164] and/or disruption of interaction of TMEM16F with the cytoskeleton [169]. In addition, during recordings in the inside-out patch-clamp configuration, the TMEM16F current is rapidly activated as soon as the patch is excised and exposed to a solution supplemented with Ca^2+^ [128,143,145,170], while during whole-cell recordings the TMEM16F current gradually increases to reach a steady-state level within minutes [8,142,145]. In contrast, large heterologous TMEM16A and B currents are detected as soon as the whole-cell configuration is established [1,18,22,43,54,133].

A range of divalent cations may lead to activation of TMEM16x channels, but with different apparent EC_50_ (Ca^2+^ > Sr^2+^ ≈ Ni^2+^ > Ba^2+^) [54,100,141,171] (Figure 4, Table 1). The Hill coefficients of the dose–response curves for Ca^2+^, Sr^2+^, and Ba^2+^ vary from ~2.5 for Ca^2+^ to ~1.5 for Sr^2+^ and Ba^2+^, possibly indicating different degrees of cooperativity for these ligands [141]. These divalent cations may bind to the principal Ca^2+^-binding pocket; thus, in the presence of saturating [Ca^2+^]_i_, the addition of Sr^2+^, Ni^2+^, and Ba^2+^ produces no further current activation [171]. Intracellular divalent cations may also produce surface charge screening on the headgroups of phospholipids in the vicinity of the permeation pathways of TMEM16x channels, thus affecting the electrostatic potential in the proximity of the pore, which may influence ion permeation [170,172].

Other divalent cations such as Co^2+^, Zn^2+^, and Mg^2+^ (Table 1) are equivalent to competitive antagonists that lack the capacity to induce opening of the TM6 steric gate but compete with Ca^2+^ binding to the principal Ca^2+^-binding pocket, thereby producing a rightward shift in the current versus [Ca^2+^]_i_ relationship for TMEM16A [141,171,173]. Binding of divalent (e.g., Mg^2+^) or trivalent (Gd^3+^) (Table 1) cations to the principal Ca^2+^-binding pocket also produces some attenuation of the electrostatic gate and increases the conductance of the TMEM16A channel [122,123].

### 5.2. Calmodulin

The possibility that TMEM16x sensitivity to intracellular Ca^2+^ is modulated by the Ca^2+^-binding messenger protein calmodulin (CaM), which is ubiquitously expressed in eukaryotic cells, has been explored extensively, but the outcome of this research effort is still inconclusive (Figure 4, Table 1). The following observations support the proposition that CaM is not mandatory for the response of TMEM16x channels to intracellular Ca^2+^: (1) CaM had no effect on TMEM16A and B currents when applied during inside-out patch-clamp recordings [22,54,174]; (2) heterologous expression of mutant forms of CaM with reduced Ca^2+^ sensitivity did not affect the amplitude of TMEM16A [174,175] or B [175] currents; (3) co-immunoprecipitation experiments revealed weak association between TMEM16A and CaM [174,176,177]; (4) low concentrations of intracellular Ba^2+^ (which does not bind to CaM [178]) activated the TMEM16A current [174]; (5) purified TMEM16A proteins reconstituted into liposomes elicited Ca^2+^-activated currents [177]; and (6) heterologous TMEM16F currents were not affected by pharmacological inhibition of Ca^2+^/CaM-dependent protein kinase II (CaMKII) [144].

In spite of the observations described above, a series of putative CaM-binding sites on TMEM16x proteins were identified. Bioinformatics analysis led to the identification of putative CaM-binding domains (CaM-BD1 and CaM-BD2) in TMEM16A [179] (Figure 1); however, there was no experimental evidence that CaM can bind to CaM-BD2 [179]. In addition, the CaM-BD1 sequence is in part encompassed within the splice segment *b*. The notion that TMEM16A channels lacking segment *b* mediate CaCC currents [46,48,180] suggests that CaM binding at this site is not mandatory for TMEM16A channel activity. Furthermore, splice segment *b* is associated with reduced Ca^2+^ sensitivity in TMEM16A [88].

An additional putative CaM-binding site, termed regulatory calmodulin-binding motif (RCBM), was identified in the N-terminus of TMEM16A and B channels, and Ca^2+^-CaM was shown to bind to isolated RCBM [175] (Figure 1). More recently, Jung et al. [181] reported two CaM-binding motifs (CBM1 and CBM2), with CBM1 partly overlapping with the CaM-BD1 described in [179] (Figure 1). Application of CaM to excised patches enhanced the HCO_3_^−^ permeability at high [Ca^2+^]_i_ in the acTMEM16A splice variant [181], although another study showed that in equimolar Cl^−^ conditions exogenous CaM did not alter TMEM16A anion permeability [174].

Native CaCC currents in arterial smooth muscle cells are inhibited by CaMKII; thus, CaCC current activation can be evoked by (1) treatment with generic inhibitors of phosphorylation (synthetic AMP-PNP), (2) omission of ATP in the intracellular solution, or (3) gene silencing with siRNA directed against CaMKII [161,182,183,184] (Figure 4, Table 1); the former two types of treatment also reduce heterologous TMEM16A current rundown in HEK-293 cells [74,179,185]. Dephosphorylation of native CaCCs in isolated rabbit pulmonary arterial smooth muscle cells (PASMCs) may lead to an increase in current density. Dephosphorylation was induced via intracellular dialysis of a solution deprived of ATP and supplemented with KN-93 (a CaMKII inhibitor) during whole-cell patch-clamp recordings [186]. In isolated PASMCs, CaMKII affected CaCCs’ gating in response to depolarising V_m_ steps, and produced a rightward shift in the V_m_ dependency of the current [182]. Noise analysis of heterologous TMEM16A currents revealed that single-channel current (*i*) was significantly reduced by CaMKII phosphorylation, while the maximum open probability (*P_o_*) and channel number (*N*) were unaffected [104].

CaMKII (γ isoform) may physically interact with heterologous acTMEM16A channels and catalyse phosphorylation of (1) S673 in proximity to the principal Ca^2+^-binding pocket, and (2) S471, in the first intracellular loop [104,183,185]. Phosphorylation of these residues leads to current inhibition [104,183,185]. In contrast to the role of CaMKII in arterial smooth muscle cells and heterologous expression systems, CaMKII (β isoform) may enhance the surface expression and TMEM16A channel activity in a glioblastoma cell line and promote cell migration and invasion [187].

### 5.3. Intra- and Extracellular pH

Acidification of intracellular pH (pH_i_) below 6 favours protonation of residues (e.g., N650, E654, E702, E705, E734, and D738 in acTMEM16A) that form the principal Ca^2+^-binding pocket of TMEM16A [188,189]. Thus, H^+^ effectively competes with Ca^2+^ for the same site, leading to apparent current inhibition (Figure 4, Table 1), as illustrated by the observations that (1) at saturating [Ca^2+^]_i_ the inhibitory effect of intracellular H^+^ ceased [189], and (2) amino acid substitution of the residues forming the principal Ca^2+^-binding pocket led to reduction in the sensitivity of the TMEM16A channel to intracellular H^+^ (N650A/E654Q (mild effect), E702Q/E705Q (strong effect), and E734Q/D738N (strong effect)) [188]. The inhibition of TMEM16A by intracellular H^+^ may constitute a protective feedback mechanism in some epithelial cells [188]. In pancreatic acinar cells, the HCO_3_^−^ efflux through TMEM16A channels contributes to pH balance in the lumen of the acini in face of the acidification caused by the release of zymogen granules within the acinar lumen [190,191]; in turn, the HCO_3_^−^ efflux leads to intracellular acidification and TMEM16A inhibition in acinar cells [191], constituting a negative feedback loop that prevents excessive loss of HCO_3_^−^ in the acinar cells [188].

Another study suggested that intracellular H^+^ may effectively work as a low efficacy agonist, which may contribute to the presence of a TMEM16A current at highly depolarised V_m_ in the absence of intracellular Ca^2+^ (see also, Section 5.1) [192]. This is because titration of the acidic residues in the principal Ca^2+^-binding pocket of TMEM16A is favoured at depolarised V_m_; the resulting protonation of the residues of the Ca^2+^-binding pocket may trigger channel opening and, possibly, attenuation of the electrostatic gate [192]. Indeed, the substitution of the acidic residues of the principal Ca^2+^-binding pocket with glutamines, mimicking a permanent protonation, allowed the V_m_ gating at physiological pH_i_, even with 0 [Ca^2+^]_i_ [192].

The TMEM16F protein is also affected by changes in pH_i_. Liang and Yang [189] reported that pH_i_ < 7 reduced both TMEM16F channel and scramblase activities, while pH_i_ > 8 potentiated these functions. The activation of the TMEM16F channel at low pH_i_ was detected at [Ca^2+^]_i_ < 15 μM, while at higher [Ca^2+^]_i_ the effect of low pH_i_ became less prominent, suggesting that intracellular Ca^2+^ and H^+^ compete for the same site [189]. Substitution of E667 with glutamine within the principal Ca^2+^-binding pocket of TMEM16F shifted the peak of [Ca^2+^]_i_, which reduced TMEM16F pH_i_ sensitivity from 15 μM to 3 mM [189]. In contrast, alanine substitution of D859 and E395 within the auxiliary Ca^2+^-binding site located on TM2-TM10 did not affect the sensitivity of TMEM16F to intracellular H^+^, suggesting no role for this site in controlling pH_i_ sensitivity [189]. The pH_i_-mediated modulation of TMEM16F channel and scramblase functions may be relevant in the pathophysiology of cancer, since dysregulated pH is a hallmark of tumour progression [193]. TMEM16A and F are upregulated in several cancer types [34,194]. The notion that these TMEM16x members are capable of sensing changes in pH_i_ may provide new insights into the involvement of TMEM16x in carcinogenesis and tumour progression.

The TMEM16A channel is also controlled by extracellular H^+^ [195] (Figure 4, Table 1). The channel is activated when extracellular pH (pH_o_) reaches values below 7. A glutamate (E623 in acTMEM16A) that is located in the extracellular loop connecting TM5 and TM6 is crucially involved in this response [195] (Figure 1). Non-stationary noise analysis revealed that titration of E623 by extracellular H^+^ allosterically increases the *P_o_* of the TMEM16A channel, without altering the single-channel conductance, thus enabling activation of TMEM16A at non-saturating [Ca^2+^]_i_—possibly through a conformational rearrangement that stabilises the principal Ca^2+^-binding pocket [195]. The residue is highly conserved in different TMEM16x homologues and paralogues; thus, it may also confer H^+^ sensitivity in other TMEM16x proteins [195]. Modulation of TMEM16A by extracellular H^+^ could be important in pathophysiological conditions, since changes in pH_o_ occur during cellular injury, ischaemia, or tumour progression [196] in the brain [197], retina [198,199,200], or epithelial cells [201,202,203,204,205,206,207], where CaCC channels are expressed [17,46,208].

### 5.4. Intra- and Extracellular ATP

Adenosine triphosphate (ATP) is an important extracellular signalling molecule in several epithelia, and serves as a neurotransmitter in both the peripheral and central nervous systems. Extracellular ATP binds to purinergic P2Y (G_q_-protein-coupled receptors) and P2X (non-selective cation channels) receptors, leading to an increase in [Ca^2+^]_i_ that may promote activation of TMEM16x scramblases [209] and/or TMEM16x channels in airway epithelial cells [210,211,212,213,214], colonic epithelial cells [215], murine taste cells [216], supporting cells of the murine olfactory epithelium [217], and arterial smooth muscle cells [180].

Intracellular ATP may also modulate TMEM16A activity, as exemplified by the observation that the inclusion of apyrase, an ATP-cleaving enzyme, in the pipette solution during whole-cell patch-clamp recordings dampened heterologous abcTMEM16A and acTMEM16A channel activity [179]. Intracellular ATP may indirectly induce TMEM16A current activation by serving as a substrate for the synthesis of PIP_2_ and/or CaMKII-mediated phosphorylation of serine residues in TMEM16A [104,218,219]. Other TMEM16x proteins are also influenced by intracellular ATP (Figure 4, Table 1). Intracellular MgATP, but not Na_2_ATP, prevented TMEM16F current rundown in inside-out patches, mimicking the activating effect of PIP_2_ [164]. Whole-cell TMEM16F current rundown could also be prevented by MgATP-promoted cytoskeletal actin polymerisation [169]. Somewhat counterintuitively, disruption of the actin cytoskeleton with cytochalasin-D [169] or as a result of patch excision [145,169] hastened the activation kinetics of the TMEM16F current, while actin-filament-stabilising agents inhibited TMEM16F activity [169].

Reduced cytoplasmic concentration of ATP during altered metabolic states—for example, during cell hypoxia and ischaemia—has the potential to reduce the extrusion of Ca^2+^ from the cytoplasm (e.g., via the plasma membrane Ca^2+^-ATPase) and, thus, lead to an increase in [Ca^2+^]_i_ and the activation of TMEM16x. It is plausible that this mechanism may constitute a relevant aspect of cellular responses to ischemia, and participate for example in arteriolar and pericyte contraction that may occur during cerebral ischaemia, given that the TMEM16A channel forms a crucial depolarising force in contractile vascular cells [220,221].

### 5.5. Hypoxia and Reactive Oxygen Species (ROS)

Tissue hypoxia is associated with conditions such as stroke, angina pectoris, myocardial infarction, heart failure, and peripheral artery disease. Hypoxia has been shown to enhance TMEM16A current density in cultured murine cardiac vascular endothelial cells [222] (Figure 4, Table 1). The underlying mechanism is an increase in TMEM16A expression, including alteration of the repertoire of the splicing variants being expressed [222]. Chronic hypoxic pulmonary hypertension is associated with enhanced TMEM16A current density and increased levels of TMEM16A mRNA and protein expression in murine PASMCs [223]. These effects may lead to vessel contraction and remodelling [223]. TMEM16A is also upregulated in idiopathic forms of pulmonary hypertension in humans [224]. Long-term hypoxia can also enhance TMEM16A expression and current density in epithelia, such as in cultured sinonasal epithelial layers [225].

In an airway-epithelium-derived cell line, hypoxia-induced augmentation of reactive oxygen species (ROS)—and the consequent peroxidation of plasma membrane lipids—increased TMEM16F activity [152]. This resulted in PS (phosphatidylserine) exposure, inflammatory cell death, and apoptosis [226] (Figure 4, Table 1). Hypoxia, oxidative stress, and lipid peroxidation may also activate TMEM16A during conditions such as polycystic kidney disease [227]. The underlying mechanism likely involves store-operated Ca^2+^ entry triggered by oxidative stress [227]. Lipid peroxidation induced by ROS enhances heterologous TMEM16A and F currents and activates TMEM16F-mediated phospholipid scrambling in HEK-293 cells [228].

### 5.6. Heat

The TMEM16x channel activity is highly sensitive to heat [228,229,230,231] (Figure 4, Table 1). The thermal sensitivity of TMEM16A becomes prominent at temperatures > ~37 °C, with an apparent temperature coefficient (*Q*_10_) of ~20. The temperature threshold for TMEM16A activation is lowered as [Ca^2+^]_i_ is increased [230]. At temperatures > 44 °C, small heterologous TMEM16A currents can be detected even at 0 [Ca^2+^]_i_ [230]. The underlying biophysical mechanism of TMEM16A heat activation is not fully defined; it may involve protein domains serving as specific thermal sensors—as suggested for other strongly temperature-sensitive channels, such as TRP channels [232,233,234]—or it may arise from the difference in heat capacity between open and closed states [235]. The increases in [Ca^2+^]_i_ and temperature have a synergistic effect on TMEM16A activation; thus, concomitant increases in these variables stimulate the TMEM16A channel more substantially than either factor alone [230]. Furthermore, the heat-mediated activation can be observed even at saturating [Ca^2+^]_i_ [17,230]. It remains to be established whether heat promotes changes in *P_o_* and/or affects *i* or *N*.

In virtue of its thermal sensitivity and high expression in dorsal root ganglion (DRG) neurons, the TMEM16A channel plays a key role in nociceptive thermal sensitivity [230,236]. Consistently, DRG neurons from mice in which the *Tmem16A* gene was deleted (knockout) had reduced heat-activated Cl^−^ currents [230,237]. A role for TMEM16A in nociception was also demonstrated by the observation that the channel is activated in response to bradykinin, which is released at sites of tissue damage and inflammation [237]. Behavioural experiments demonstrated that pharmacological inhibition of TMEM16A (with mefloquine or with small interfering RNA (siRNA)) or *Tmem16A* knockout impaired the normal response to heat in tail-withdrawal tests [230]. TMEM16B, but not TMEM16D or E, is also temperature sensitive [230,238]. However, a major role for TMEM16B in thermal nociception was excluded because it is expressed in DRG neurons at very low levels compared to TMEM16A [230].

TMEM16F proteins are also markedly heat sensitive, and demonstrate increased Ca^2+^ sensitivity during whole-cell patch-clamp recordings at 37 °C relative to the activity measured at room temperature [228]. All TMEM16F splice variants are activated at temperatures between 37 and 42 °C in the submicromolar range of [Ca^2+^]_i_ [231]. However, the TMEM16F current amplitude was not significantly increased by temperatures > 42 °C, in contrast with what was observed for the TMEM16A channel [230,231]. The temperature sensitivity of TMEM16F scramblase function remains not fully defined, having been reported to either decrease [231] or increase [239] at 37 °C relative to measurements of scramblase activity at room temperature.

### 5.7. Lipids

#### 5.7.1. PIP_2_

As noted above (see Section 3), TMEM16x proteins are activated by G_q_PCR stimulation. The associated activation of PLC leads to PIP_2_ breakdown and the formation of IP_3_, which promotes an increase in [Ca^2+^]_i_ and TMEM16x activation. PIP_2_ binds to cloned and native smooth muscle TMEM16A channels in membrane extracts [240]. Ta et al. [241] investigated whether PIP_2_ controls the function of TMEM16A and B channels. Cloned acTMEM16A was activated by a water-soluble PIP_2_ analogue during inside-out patch-clamp recordings. Depletion of endogenous PIP_2_ with a genetically encoded *Danio rerio* voltage-sensitive phosphatase (DrVSP) reduced heterologous TMEM16A currents [241]. This effect was attenuated by an inactivating mutation in DrVSP and antagonised by co-expression of a phosphatidylinositol-4-phosphate 5-kinase that catalyses PIP_2_ formation. In contrast, the TMEM16B channel was inhibited by PIP_2_ [241]. The effect of PIP_2_ on TMEM16A channels was V_m_-independent, and was especially pronounced in the low micromolar range of [Ca^2+^]_i_ (<~2 μM). In contrast, the effect of PIP_2_ on TMEM16B did not differ significantly over a wide range of [Ca^2+^]_i_, but was only detectable at highly depolarised V_m_ (≥50 mV) [241]. PIP_2_ affected TMEM16A and B current amplitude via modulation of *P_o_*, while *i* and ion selectivity remained unaltered [241]. It was proposed that in vivo PIP_2_ may modulate TMEM16A under resting conditions, as well as during membrane depolarisation, to constitute a negative feedback on G_q_PCR-mediated TMEM16A current activation by [Ca^2+^]_i_ [241]. In contrast, TMEM16B may be modulated only at highly depolarised V_m_, which might be reached by some types of excitable cells during action potential firing—especially during pathological conditions associated with elevations of the action potential peak, such as hypernatremia [241].

Other groups also reported that PIP_2_ activated cloned TMEM16A channels (Figure 4, Table 1) and prevented current rundown during recordings in excised patches [166,167,215]. In *Xenopus laevis* oocytes, depletion of membrane PIP_2_ through applications of a PIP_2_-sequestering agent (neomycin) resulted in inactive TMEM16A channels even in the presence of saturating [Ca^2+^]_i_, suggesting that PIP_2_ and Ca^2+^ are both mandatory for TMEM16A function [219]. Native CaCC currents in rodent PASMCs, which are mediated by TMEM16A [48], were found to be inhibited by PIP_2_ [240]. In addition, internal application of water-soluble PIP_2_ analogues in whole-cell recordings produced little-to-no effect on endogenous TMEM16A currents in HT_29_ colonic epithelial cells [215]. Thus, the mechanism of PIP_2_ regulation of native CaCCs in mammalian cells may not be univocal, and may vary depending on cell-type-specific regulatory components.

Regulation of the TMEM16A channel by PIP_2_ may vary depending on the splice variant, with heterologous acTMEM16A displaying higher channel activity and more pronounced current rundown following PIP_2_ depletion than aTMEM16A [104]. PIP_2_ resynthesis, dependent on cytosolic ATP, was essential for acTMEM16A recovery from rundown [218,241]. Mutagenesis and atomistic simulations suggested that CaMKII phosphorylation of S673 (in acTMEM16A) in the third intracellular loop reduced the channel’s sensitivity to PIP_2_ [104]. TMEM16A splice variants lacking the segment *c* were less sensitive to S673 phosphorylation [104].

Le et al. [167] identified a putative PIP_2_-binding site in aTMEM16A composed of six basic residues (three arginine and three lysine) at the cytosolic interface of TM3–TM5. Binding of PIP_2_ at this site stabilised the open state and prevented current rundown [167] (Figure 1). The ion-conducting pore of TMEM16A therefore consists of two functionally distinct modules: the principal Ca^2+^-binding pocket module formed by residues in TM6–TM8, and the PIP_2_-binding site regulatory module involving residues in TM3–TM5 [167] (Figure 1). It was suggested that Ca^2+^ and PIP_2_ synergistically promote TMEM16A activation [167]. The possibility that other members of the family may share the dual regulation by Ca^2+^ and PIP_2_ observed in TMEM16A could not be excluded [167]. Using unbiased atomistic MD simulations and single-point mutagenesis, Yu et al. [242] identified eight additional putative interaction sites for PIP_2_ on acTMEM16A. Maximal activation of TMEM16A requires interaction with both PIP_2_ and Ca^2+^ [218,242,243].

The experimentally determined structure of the open TMEM16A channel is not yet available. Using atomistic simulations, Jia and Chen [137] obtained a model of the open channel. Binding of PIP_2_ to TMEM16A was sufficient to induce spontaneous dilation of the pore through a rearrangement of TM3 and TM4, similarly to another open-channel model proposed by a different group [122] (Figure 2). These studies provide insights into the conformational changes triggered by PIP_2_ binding, as well as the conformation of the open TMEM16A channel.

The TMEM16F channel/scramblase is also activated by PIP_2_ [164]. TMEM16F’s Ca^2+^ response is desensitised by a brief exposure to high [Ca^2+^]_i_, but subsequent exposure to PIP_2_ or water-soluble PIP_2_ analogues restores TMEM16F channel activity [164]. Mutagenetic analysis revealed that electrostatic interactions of PIP_2_ with a cluster of positively charged amino acids within the N-terminus modulate TMEM16F synergistically with V_m_ depolarisation to facilitate Ca^2+^ gating [164] (Figure 1).

#### 5.7.2. Other Lipids

Plasmalemmal lipids can serve as both substrates and modulators of scramblase activity and ion conduction in afTMEM16, nhTMEM16, and mammalian TMEM16x scramblases [156,157] (Table 1). For example, in liposomes composed of 1-palmitoyl-2-oleoyl-*sn*-glycero-3-phosphatidylethanolamine/1-palmitoyl-2-oleoyl-*sn*-glycero-3-phosphatidylglycerol (POPE/POPG), afTMEM16 and nhTMEM16 had reduced ion transport rates, which were enhanced in liposomes supplemented with 1-palmitoyl-2-oleoyl-*sn*-glycero-3-phosphatidylcholine (POPC) [139,244]. Liposomes composed of POPC/POPG led to reduced scrambling activity of nhTMEM16 [111].

The primary structure of TMEM16A encompasses 14 potential cholesterol-binding motifs, but only the site near the extracellular end of TM5 has been shown—via computational docking—to bind cholesterol [166]. Exposure of isolated murine portal vein myocytes to methyl-β-cyclodextrin (M-βCD)—an agent that depletes membrane cholesterol—increased the CaCC current amplitude (Figure 4, Table 1) without affecting other channel properties, such as the sensitivity to NFA and A9C (generic Cl^−^ channel blockers) [245]. Acute application of M-βCD also enhanced THE whole-cell TMEM16A current heterologously expressed in HEK-293T cells [166].

A number of fatty acids, including some dietary polyunsaturated fatty acids (PUFAs), inhibit TMEM16A channel activity (Figure 4, Table 1). These include saturated stearic acid (SA), uncharged methyl stearate (Me-S), monounsaturated oleic acid (OA), polyunsaturated arachidonic acid (AA), polyunsaturated eicosapentaenoic acid (EPA), and polyunsaturated docosahexaenoic acid (DHA) [166]. The underlying mechanism (gating modification or direct occlusion of the pore) remains undefined [166]. The effect of EPA and OA (poly- and monounsaturated long-chain fatty acids, respectively) was detectable only at positive V_m_ (120 mV), while DHA (a polyunsaturated long-chain fatty acid) exhibited current inhibition even at negative V_m_ (−100 mV) [166]. The mechanism of V_m_ dependence of inhibition did not rely on the charge of the lipid headgroup, since uncharged saturated fatty acid analogues of OA also inhibited TMEM16A in a V_m_-dependent fashion, albeit to a lesser extent than OA [166]. Thus, the mechanism underlying the V_m_ dependence of fatty acid inhibition may be secondary to V_m_-dependent conformational changes in TMEM16A. The saturated SA produced a much smaller effect in comparison with unsaturated fatty acids with one or more double bonds [166]. The number of double hydrogen bonds in *cis* geometry in the fatty acid tail is a determinant of fatty acid modulation of several ion channels [246], and may also be a determinant of TMEM16A inhibition.

TMEM16A regulation by lipids may also be important in pathology. Whole-cell TMEM16A currents increased in a murine lung adenocarcinoma cell line (LA795) treated with 10 µg lipopolysaccharide (LPS) (Table 1) for 24 h [247]; this appears to have been due to an increase in TMEM16A mRNA and protein levels after LPS exposure [247]. LPS treatment (10 μg/mL LPS, 24–36 h) induced TMEM16A overexpression in adenocarcinomic human alveolar basal epithelial cells (A549). The underling mechanism remains not fully defined, but TMEM16A activation was associated with reductions in tumour necrosis factor-α (TNF-α) and interleukin 8 (IL-8) secretions, as well as inhibition of the ‘nuclear factor kappa light-chain enhancer of activated B cell’ (NF-κB) pathway [247,248]. TMEM16A expression in rat intestinal epithelial (RAW264.7) cells was increased by exposure to high doses of LPS (10 µg/mL, 12–36 h) [249]. Treatment of monolayers of a rat intestinal epithelial cell line (EEC-6) with low doses of LPS (0.1–1 µg/mL) worsened cell barrier dysfunction, possibly by activating extracellular-signal-regulated kinase1/myosin light-chain kinase (ERK1/MLCK) signalling pathways [249]. However, treatment with higher doses of LPS (10 µg/mL) resulted in a protective effect mediated by TMEM16A activation, and possible subsequent recruitment of the extracellular signal-regulated kinase/B-cell lymphoma-2/Bcl-2-associated x protein (ERK/Bcl-2/Bax) signalling pathways via an undefined mechanism [249]. Collectively, these studies might imply that TMEM16A could play a role in the pathology of LPS-induced inflammation. However, the concentration of LPS used in these studies was significantly higher than the plasma LPS levels detected in human patients during severe sepsis [250]; thus, the pathophysiological significance of the above observations remains unclear.

Phospholipids such as lysophosphatidic acid (LPA) (Figure 4, Table 1) and arachidonic acids (AAs) (Table 1) are also modulators of TMEM16x function. Acutely applied AA inhibited heterologous TMEM16F current during whole-cell patch-clamp recordings [251]. Pharmacological modulation of phospholipase A2 (PLA_2_) impacted heterologous abcTMEM16A and V1-TMEM16F currents and PS scrambling by V1-TMEM16F in HEK-293 cells [228]. Heterologous TMEM16F current is enhanced by stimulation of PLA_2_ through generation of plasma membrane LPA, independent of any increase in [Ca^2+^]_i_ [251]. Lysophospholipids (LPL), such as LPA, may affect cell membrane tension [252] and possibly promote scramblase activity, even in the absence of Ca^2+^ [251]. LPA reaches a plasma concentration of up to ~10 µM [253,254] during activation of platelets [255,256]. LPA triggers an increase in [Ca^2+^]_i_ by promoting opening of Ca^2+^ channels within the membranes of erythrocytes [257]. The [Ca^2+^]_i_ increase provokes TMEM16F-mediated exposure of PS on the outer surface of the erythrocyte membrane that promotes the thrombogenic process and blood clotting [70,258,259,260,261]. Natural products such as tannic acid (TA) and epigallocatechin-3-gallate (EGCG) inhibit TMEM16F-mediated PS exposure induced by LPA [226,239,261,262]. However, a direct effect of TA and EGCG on TMEM16F scramblase activity has been questioned, since these compounds act as fluorescence quenchers [263]; this results in loss of fluorescence emission of annexin V—a commonly used PS-binding probe to report scrambling activities [263]. The studies above thus suggest that the downstream products of PLA_2_ may have contrasting effects on TMEM16F activity, with AA and LPL causing inhibition or activation of TMEM16F currents, respectively [228,251].

TMEM16A is activated by bile acid uptake in the apical cholangiocyte membrane of murine, rat, and human biliary epithelium, and may promote increased bile flow during cholestasis through ductular secretion [264]. TMEM16A-mediated Cl^−^ transepithelial secretion is rapidly increased by intracellular dialysis of ursodeoxycholic acid (UDCA) or tauroursodeoxycholic acid (TUDCA) (Table 1) [264]. The extracellular application of both UDCA and TUDCA induced exocytosis, ATP release, and an increase in [Ca^2+^]_i_, resulting in the activation of TMEM16A currents [264]. TMEM16A currents were not observed when UDCA and TUDCA were applied together with inhibitors of P2Y and IP_3_ receptors, such as apyrase, suramin, or 2-aminoethoxydiphenyl borate (2-APB) [264]. Bile acid activates TMEM16A currents via purinergic signalling that mediates an increase in [Ca^2+^]_i_ through IP_3_-receptor-dependent Ca^2+^ release from intracellular stores [264]. Thus, bile acid released by hepatocytes activates TMEM16A currents in the downstream cholangiocytes, which may represent an example of hepatobiliary coupling that links bile acid release with modulation of ductular Cl^−^ transport [264].

## 6. Other Regulators of TMEM16x Activity

### 6.1. Ca^2+^-Activated Chloride Channel Regulators 1 and 2 (CLCA1 and 2)

The Ca^2+^-activated chloride channel regulators (CLCAs) [265] are a family of secreted self-cleaving metalloproteases that modulate CaCC activity in mammalian cells [266]. CLCAs play important roles in mucus homeostasis [267], and their dysfunction is implicated in pathologies such as asthma and chronic obstructive pulmonary disease (COPD) [268]. Overexpression of CLCAs leads to CaCC activation in a variety of cell types [269,270,271,272], and CLACs were initially proposed as CaCCs’ pore-forming subunits [265].

Secreted CLCA1 modulates TMEM16A channels in a paracrine fashion by increasing TMEM16A surface expression, stabilising the TMEM16A homodimer configuration and promoting channel activity through a physical interaction with TMEM16A [273] (Figure 4, Table 1). CLCAs may also stimulate store-operated entry of Ca^2+^, and the resulting change in [Ca^2+^]_i_ may affect TMEM16A activity [274] (Figure 4, Table 1). CLCA2 interacts with the store-operated Ca^2+^ channel ‘calcium-release-activated calcium channel protein 1′ (ORAI-1) and the ER calcium sensor ‘stromal interaction molecule 1′ (STIM-1) in HEK-293 cells stably expressing human CLCA2 [274]. TMEM16A and CLCA2 are upregulated in some tumours, which may lead to enhancement of TMEM16A currents and tumour progression [274,275,276,277].

A secreted form of CLCA1, the N-terminal CLCA1 (N-CLCA1), encompasses a von Willebrand factor type A (VWA) domain that is involved in the interaction with TMEM16A and activation of TMEM16A currents [273,278]. Injection of N-CLCA1 into the trachea of mice produced intraluminal mucus accumulation in the airways, possibly through increased expression of TMEM16A channels in the apical membranes of airway epithelial cells [279].

### 6.2. KCNE1

KCNE1 is a single-TM-domain protein that forms an ancillary (β) subunit of the V_m_-gated K^+^ channel KCNQ1 [280,281]. Ávalos Prado et al. [282] suggested that KCNE1 also serves as an auxiliary subunit of the TMEM16A channel, and that it assembles with a 2:2 stoichiometry. In the presence of KCNE1, the TMEM16A channel was found to be active at positive V_m_ even with 0 [Ca^2+^]_i_ [282]. It was proposed that KCNE1 modulates TMEM16A activity by favouring TM6 (steric gate) opening [282] (Figure 4, Table 1). A region of 13 residues was identified as the minimal portion of KCNE1 sufficient to produce modulation of the TMEM16A current [282]. Moreover, mutant forms of KCNE1 harbouring single-nucleotide polymorphisms—such as S38G and R32H, which are associated with predisposition to heart failure and long QT syndrome (LQTS) in human subjects—failed to regulate TMEM16A currents [282]. This observation possibly implicates KCNE1 regulation of TMEM16A in the onset of these pathologies. KCNE5, a KCNE1 homologue, also physically interacts with TMEM16A [282]. Unlike TMEM16A, the TMEM16B channel appeared not to be regulated by KCNE1 or KCNE5 [282].

## 7. Additional Regulatory Mechanisms of TMEM16x Proteins

TMEM16x proteins are subjected to post-translational modifications such as protein phosphorylation (reviewed in [99] for the TMEM16A channel). TMEM16x proteins may also be subjected to SUMOylation (covalent attachment of a small ubiquitin-related modifier (SUMO) to a protein), with SUMOylation sites having been identified in TMEM16B, but not TMEM16A [283]. It is currently unknown whether TMEM16x proteins are subject to RNA editing, a form of post-transcriptional modification that applies to a range of ion channels [284].

## 8. A Role for TMEM16F in the Pathology of SARS-CoV-2

TMEM16F may also play a role in the pathogenesis of SARS-CoV-2. A characteristic feature of SARS-CoV-2 infection is the formation of pneumocyte syncytia, which involves viral spike protein cleavage by host cell proteases [285,286]. Cells expressing SARS-CoV-2 spike proteins have enhanced Ca^2+^ oscillations and increased TMEM16F activity, leading to PS externalisation, which promotes syncytia formation [29]. SARS-CoV-2 spike proteins may (1) promote Ca^2+^ release and, thus, activation of TMEM16F scrambling in infected cells (*cis* modality), and/or (2) stimulate activation of proteases on neighbouring cells, triggering cell fusion (*trans* modality) [29]. The involvement of TMEM16F in SARS-CoV-2 spike-induced syncytia is supported by the role of PS exposure in a range of other physiological cell fusion events [287,288,289,290,291]. Niclosamide—a therapeutic anthelmintic drug utilised for the treatment of tapeworm infections—reduces syncytial formation by inhibiting TMEM16F activity, and could be repurposed for the treatment of SARS-CoV-2 [29,292].

## 9. Conclusions

The TMEM16x family of Ca^2+^-activated channels and scramblases provides a link between intracellular Ca^2+^ handling and ion/lipid transport. It is becoming apparent that these proteins respond to a range of additional cellular factors, highlighting their capacity to serve as sensors of cellular homeostasis. Uncovering these intricate mechanisms of regulation will enable a more complete understanding of the TMEM16x physiological roles and aid in the exploitation of these potential pharmacological targets for the treatment of a range of human diseases, including stroke, hypertension, vascular dementia, cystic fibrosis, and cancer.

## Figures and Tables

**Table 1:** List of the key factors controlling TMEM16x activities.

Modulator	TMEM16x	Effect
Ca^2+^, Sr^2+^, Ni^2+^, Ba^2+^	CaCCs, A, B, F	Agonist
Co^2+^, Zn^2+^, Mg^2+^	A	Antagonist
Mg^2+^, Gd^3+^	A	Charge screening
Calmodulin	A, B	Modulation of ion channel properties (contrasting results)
CaMKII	CaCCs, A	Current inhibition
pH_i_	F	Inhibition of channel and scramblase activities
pH_i_	A	Current modulation
pH_o_	A	Current activation
ATP	A, F	Current activation/reduction of current rundown (contrasting effects)
Hypoxia	A	Current activation
ROS	A, F	Activation of scramblase and channel activities
Heat	A, F	Current activation; Modulation of scramblase activity
PIP_2_	A, F	Activation of channel and scramblase activities
PIP_2_	CaCCs, B	Current inhibition
Plasmalemmal lipids	afTMEM16, nhTMEM16	Contrasting effects
Cholesterol	CaCCs, A	Current inhibition
Fatty acids	A	Current inhibition
LPS	A	Increase in current density
LPA	A, F	Activation of channel and scramblase activities
AA	F	Current inhibition
Bile acids (UDCA and TUDCA)	A	Current activation
CLACs	CaCCs, A	Increase in current density
KCNE1	A	Increase in current density
